# The impact of phosphate-balanced crystalloid infusion on acid-base homeostasis (PALANCE study): study protocol for a randomized controlled trial

**DOI:** 10.1186/s13063-017-2051-z

**Published:** 2017-07-10

**Authors:** Judith-Irina Pagel, Nikolai Hulde, Tobias Kammerer, Michaela Schwarz, Daniel Chappell, Alexander Burges, Klaus Hofmann-Kiefer, Markus Rehm

**Affiliations:** 1 Department of Anaesthesiology, Hospital of the University of Munich LMU, Marchioninistr. 15, 81377 Munich, Germany; 2 Department of Anaesthesiology, Surgical Clinic of Munich-Bogenhausen, Munich, Germany; 3 Department of Gynaecology, Hospital of the University of Munich, LMU, Munich, Germany

**Keywords:** Phosphate, Fluid replacement therapy, Weak acids [A^-^], Stewart concept, Acid-base balance

## Abstract

**Background:**

This study aims to investigate the effects of a modified, balanced crystalloid including phosphate in a perioperative setting in order to maintain a stable electrolyte and acid-base homeostasis in the patient.

**Methods/design:**

This is a single-centre, open-label, randomized controlled trial involving two parallel groups of female patients comparing a perioperative infusion regime with sodium glycerophosphate and Jonosteril® (treatment group) or Jonosteril® (comparator) alone. The primary endpoint is to maintain a stable concentration of weak acids [A^-^] according to the Stewart approach of acid-base balance. Secondary endpoints are measurement of serum phosphate levels, other acid-base parameters such as the strong ion difference (SID), the onset and severity of postoperative nausea and vomiting (PONV), electrolyte levels and their excretion in the urine, monitoring of renal function and glycocalyx components, haemodynamics, amounts of catecholamines and other vasopressors used and the safety of the infusion regime.

**Discussion:**

Perioperative fluid replacement with the use of currently available crystalloid preparations still fail to maintain a stable acid-base balance and experts agree that common balanced solutions are still not ideal. This study aims to investigate the effectivity and safety of a new crystalloid solution by adding sodium glycerophosphate to a standardized crystalloid preparation in order to maintain a balanced perioperative acid-base homeostasis.

**Trial registration:**

EudraCT number 201002422520. Registered on 30 November 2010.

**Electronic supplementary material:**

The online version of this article (doi:10.1186/s13063-017-2051-z) contains supplementary material, which is available to authorized users.

## Background

The increasing importance of an adequately balanced crystalloid in terms of outcome has been shown by a range of review articles [1–4]. Current studies target to find the “ideal physiologic” crystalloid preparation, resembling plasma concentrations, maintaining electrolyte concentrations within normal range and stabilizing acid-base balance. In the beginning of the 1990s it was shown that the (excessive) administration of isotonic saline resulted in an acid-base dysbalance, a hyperchloraemic acidosis [5] that can effectively mask perfusion deficits or result in inadequate therapeutic interventions if this condition is mistaken as tissue hypoxia [6]. Furthermore, we now know that a perioperative hyperchloraemic acidosis induced by administration of saline results in a reduction of renal perfusion and/or urinary excretion [7] as well as in a higher incidence of postoperative nausea and vomiting (PONV) morbidity and mortality [3, 4, 8–11]. Imbalances in acid-base chemistry can be described with the help of (1) the descriptive Henderson-Hasselbalch equation [12], where the blood pH is related to the bicarbonate buffer system, (2) a semiquantitative method using the later introduced concepts of base excess (BE) [13] and anion gap (AG) [14] as computable factors and (3) the quantitative physicochemical approach by Peter Stewart, which has been intensively discussed in the 1990s but now has been mostly accepted to be the “gold standard” [15–17]. The advantage of Stewart’s concept is its quantitative analytical approach that permits one to diagnose and differentiate between specific forms of acid-base disturbances that conventional methods have not been able to describe before [18].

Stewart based his model on three principles: (1) the sum of all positive charges equals the sum of all negative charges (principle of electroneutrality), (2) the dissociation equilibria of all incompletely dissociated substances must always be satisfied and (3) the total mass of a non-completely dissociated substance can be calculated by summarizing the amount of dissociated and non-dissociated forms. Three components comply to these principles at all times: (1) H_2_O in its dissociated form [H^+^] and [OH^−^] (however, the plasma concentration of these ions is extremely low (approximately 10–7 mmol/l)), (2) strong ions, such as electrolytes ([Na^+^], [K^+^], [Cl^−^], [Ca^2+^], [Mg^2+^] and lactate, which are found nearly completely dissociated and can, therefore, hardly react with other substances in the human body, and (3) weak (= incompletely dissociated) substances comprised of acid-base pairs (carbonic acid/carbon dioxide, NH_3_/NH_4_^+^) as well as non-volatile plasma proteins and phosphate [16, 18]. Stewart rigorously distinguished between three *dependent variables* the pH and the concentration of hydrogen ions [H^+^] and bicarbonate [Bic^−^] that can only passively respond to alterations determined entirely by three *independent variables*, the carbon dioxide partial pressure (paCO_2_), the weak acids [A^−^] (former [A_TOT_]) and the strong ion difference (SID, see below). Stewart also employed the Henderson-Hasselbalch equation to relate respiratory acidosis/alkalosis to changes of the paCO_2_; however, according to his model, a metabolic acidosis/alkalosis cannot be explained with it [16, 18]. Contemplating the other two independent variables, Stewart proposed that electrolytes, as well as albumin and phosphate, can shift the pH; an entirely new perspective. Currently, there are a couple of formulas proposed to calculate the SID. For the present study we will use the following formulas for [A^−^] and the SID published by Figge et al. [19]:

1 $$ \mathrm{S}\mathrm{I}\mathrm{D}=\left[{\mathrm{Na}}^{+}\right]+\left[{\mathrm{K}}^{+}\right]-\left(\left[{\mathrm{Cl}}^{-}\right]+\left[{\mathrm{Lac}}^{-}\right]\right) $$

2 $$ \left[{\mathrm{A}}^{-}\right]=\left[\mathrm{Alb}\times \left(0.123\times \mathrm{pH}-0.631\right)\right]+\left[\mathrm{Pix}\left(0.309\times \mathrm{pH}-0.469\right)\right] $$

where [Na^+^] = sodium, [K^+^] = potassium, [Cl^−^] = chloride, [Lac^−^] = lactate, [Alb] = albumin in g/L and

[Pi] = phosphate in mmol/l concentrations in the serum.

Normal values for the SID and [A^−^] in human plasma at a pH of 7.4 lie around 40 meq/l and 15 meq/l, respectively. The unit milliequivalents per litre (meq/l) refers to the electric charge (e.g., 2 mmol [Na^+^] = 2 meq). Therefore, a normal SID can be calculated with formula (1) as follows:

SID = (142 meq/l) + (4 meq/l) – (105 meq/l) – (1 meq/l) = 40 meq/l.

The following relationship between SID, [A^−^] and the pH has been established:

$$ \mathrm{S}\mathrm{I}\mathrm{D}\uparrow \mathrm{and}/\mathrm{or}\left[{\mathrm{A}}^{-}\right]\downarrow \kern2.75em \to \kern2.5em \mathrm{Alkalosis} $$

$$ \mathrm{S}\mathrm{I}\mathrm{D}\downarrow \mathrm{and}/\mathrm{or}\left[{\mathrm{A}}^{-}\right]\uparrow \kern2.75em \to \kern2.5em \mathrm{Acidosis} $$

Applying this concept, it becomes evident that an increase in chloride lowers the SID, leading to the previously mentioned hyperchloraemic acidosis and that hypoalbuminemia lowers [A^−^], shifting the balance towards an alkalotic state. These new insights into the cause of different types of metabolic acid-base disorders comprise the novelty and ingenuity of Stewart’s concept.

### Trial rationale and hypothesis

Many crystalloid preparations contain a non-physiologic concentration of electrolytes. Although the unprotected term “balanced” is widely used for different crystalloid preparations by authors and manufacturers, there is currently no perfectly balanced preparation available. NaCl 0.9% w/v (isotonic saline) solution consists of 154 mmol/l (=154 meq/l) [Na^+^] and 154 mmol/l (= 154 meq/l) [Cl^−^]. The SID of 0.9% isotonic saline is, therefore, 0 meq/l. The administration of large amounts of saline will dilute the patient’s former physiologic SID resulting in acidosis. Ringer’s lactate and other balanced solutions show a SID of 27 − 36 meq/l which is much closer to physiologic values [20, 21]. This was acquired by replacing a certain amount of chloride with a metabolizable anion like lactate, acetate or maleate. Though better balanced than 0.9% isotonic saline, these solutions neither contain albumin nor phosphate and, thus, cannot prevent the dilution of [A^−^] and hence the possible onset of an alkalotic state if infused in humans. As a consequence, they are not really “balanced” in the sense of Stewart’s approach. The key aspect of the planned investigation is to test the applicability of a new phosphate-balanced crystalloid preparation based on Stewart’s concept of acid-base. The novelty of this study is the fact that sodium glycerophosphate is administered in patients undergoing major abdominal surgery not to replenish low phosphate levels as a therapeutic approach, but to stabilize them pre-emptively.

We hypothesize that the patients’ [A^−^] levels can be stabilized when adding phosphate to a standard crystalloid. Additionally, in contrast to the control group, perioperative hypophosphatemia can be avoided by the supplementation of phosphate. It will, therefore, be tested whether Jonosteril® (Fresenius Kabi AG, Bad Homburg, Germany, see below) (investigational medical product 1 (IMP1)) in combination with sodium glycerophosphate (Fresenius Kabi AG, Bad Homburg, Germany) (IMP2)) is able to maintain a more constant acid-base balance in the patient compared to Jonosteril® alone. Additionally, a reduction of PONV is possible. Both preparations are approved substances and frequently administered during daily clinical routine.

### Jonosteril®

The crystalloid Jonosteril® is routinely administered for perioperative fluid therapy at the authors’ institution. It contains electrolytes in physiologic concentrations as well as acetate (Na^+^ 137 mmol/l, K^+^ 4 mmol/l, Ca^2+^ 1.65 mmol/l, Mg^2+^ 1.25 mmol/l, Cl^−^ 110 mmol/l, acetate^−^ 36.8 mmol/l); however, it does not carry albumin or phosphate. As such, this crystalloid is capable to maintain a SID within normal range; it will, however, not be able to prevent a decrease of [A^−^] due to the occurring dilution [22]. The latter and the decrease of albumin and phosphate will most likely result in hypoalbuminemia, shifting the system towards alkalosis. Contraindications for the use of Jonosteril® are any form of hypersensitivity or allergy to the components of the crystalloid as well as hyperhidratation and hyperkalaemia, according to the manufacturer. Only the crystalloid Jonosteril® will be tested in this study, further studies will be necessary to evaluate the applicability also for other crystalloid preparations.

### Sodium glycerophosphate

Sodium glycerophosphate (Fresenius Kabi AG, Bad Homburg, Germany; concentration 1 mmol/ml, ATC-Code: B05XA) is an electrolyte supplement approved and routinely administered for the parenteral treatment of hypophosphatemia especially on intensive care units (ICUs). Phosphate is usually not routinely measured during surgical procedures and is, therefore, not substituted. Accordingly, a hypophosphatemia occurs frequently with an incidence of between 44.8% [23] and 67% [24] and remains undetected, although the unfavourable effects in terms of post-aggression metabolism are well known. The ATP- dependent metabolism strongly relies on phosphate as a substrate, especially during the postoperative phase of major cardiac and abdominal surgery [25, 26]. This leads to a high turnover of phosphate and requires parenteral substitution. Therefore, an intraoperative substitution of phosphate seems reasonable and indicated. It has been shown that an early perioperative substitution of phosphate during liver surgery had a protective effect in terms of cardiorespiratory complication and is, therefore, recommended [27, 28]. Prior to inclusion, all patients will be evaluated regarding contraindications for the use of sodium glycerophosphate (existing chronic kidney disease or renal failure, hyperphosphatemia, hypernatremia or allergic disposition towards sodium glycerophosphate or its components). If none of the above can be detected, the parenteral application of sodium glycerophosphate is uncritical. By contrast to the dosage given on ICUs [29] or according to an estimated basic daily demand of 0.2–0.5 mmol/kg bodyweight (according to the summary of product characteristics) the amount of phosphate given during this trial is low. The maximum infusion rate is 20 mmol phosphate per hour according to the manufacturer and will not be reached. It is expected that during the observation period and due to the substitution, serum phosphate levels will rise around 0.5 mmol/l [22] which will be controlled every 30 min. Since the substituted phosphate gets rapidly metabolized and excess phosphate is excreted in the urine, a severe hyperphosphatemia is not expected [30]. Before initiation of the trial, it was confirmed that Jonosteril® and sodium glycerophosphate are compatible for the intended use. Phosphate will be added to Jonosteril® immediately prior to the application to avoid any potential risk of infection.

## Outcome measures

### Primary objective

The primary objective is to compare two infusion therapy regimes, Jonosteril® + Sodium glycerophosphate (IMP1/IMP2) versus Jonosteril® (IMP1) alone, with the aim to demonstrate superiority of IMP1/IMP2 over IMP1 due to a more stable value of [A^−^] during the observation period of 120 min after the initiation of general anaesthesia.

### Primary endpoint

The primary endpoint is the calculation of [A^−^] in mmol/l according to the Stewart approach of acid-base balance (using equation (2); numerical deviation from reference baseline value around 15 meq/l) and takes place every 30 min over 120 min (time points T1 − T5).

### Secondary objectives

The secondary objectives of the trial are the influence of the distinct infusion therapy regimens on other acid-base parameters, on the onset of PONV, laboratory chemical parameters like electrolytes and their excretion in the urine, as well as hemodynamic stability, monitoring of the vascular barrier (shedding of the glycocalyx), the the use of vasopressors and the safety of the crystalloid preparation in this setting.

Secondary endpoints of the trial are:

- Serum phosphate levels (ionized in mmol/l, non-ionized in mg/dl, continuous measure from baseline T0 and serial blood draws over a 120-min time frame T1 − T5)
- Acid-base parameters: pH (numerical), paCO_2_ (mmHg), HCO_3_^−^ (mmol/l), BE (mmol/l), anion gap (mmol/l); (continuous measure from baseline T0 and serial blood draws over a 120-min time frame T1 − T5)
- Apparent SID and effective SID (both in mmol/l), Strong Ion Gap (mmol/l), albumin, lactate (ionized in mmol/l, non-ionized in mg/dl, continuous measure from baseline measures T0 and serial blood draws over a 120-min time frame T1 − T5) serum and urine analysis of electrolytes (osmolality in mosmol/l; all other in mmol/l, continuous measure on T6, change from baseline T0)
- Documentation and analysis of mean arterial blood pressure (MAP) in mmHg, heart frequency in min^-1^, demand of vasopressors (norepinephrine dose in mg/h), stroke volume variation (SVV) in %, central venous pressure (CVP) in mmHg and cardiac output in ml/min (continuous measure from baseline T0 and serial blood draws over a 120-min time frame T1–T5)
- Assessment of serum creatinine (mg/dl) and urea (mg/dl) as well as calculation of the glomerular filtration rate (ml/min) and creatinine clearance (C_cr_) in ml/min according to the Cockroft-Gault equation [31] as continuous measure on two blood draws at T0 and T6, change from baseline T0)

$$ {\mathrm{C}}_{\mathrm{cr}}=\frac{\left(140-\mathrm{Age}\ \mathrm{in}\ \mathrm{years}\right) \times \mathrm{Mass}\ \left(\mathrm{in}\ \mathrm{kg}\right) \times \left[0.85\ \mathrm{if}\ \mathrm{female}\right]}{72 \times \mathrm{Serum}\ \mathrm{Creatinine}\ \left(\mathrm{in}\ \mathrm{mg}/ dl\right)} $$

- Monitoring of the integrity of the vascular barrier function via detection of hyaluronic acid and syndecan-1 (shedding of glycocalyx components) (in ng/ml, continuous measure from baseline T0 and serial blood draws over a 120-min time frame T1–T5)
- Evaluation and documentation of postoperative nausea and vomiting (descriptive evaluation on day of surgery until postoperative day 3, patients’ account and anaesthesia protocol of surgery, dichotomic measure: yes/no)
- Onset frequency and intensity of unexpected events: UE/ SUE and SUSARs (critical assessment on day of surgery T1–T5 until postoperative day 3, onset: date, severity score 1–3: mild, moderate, severe, according to detailed injury reporting plan)
- Necessity of renal replacement therapy (descriptive evaluation, after surgery until postoperative day-3 dichotomic measure: yes/no)

## Methods/design

**Fig. 1:**
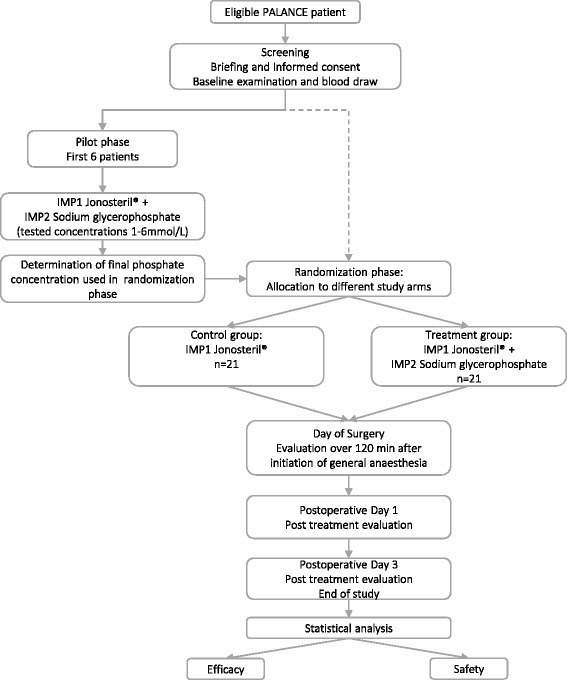
Participant flow diagram showing the organizational structure and different study groups of PALANCE

**Fig. 2:**
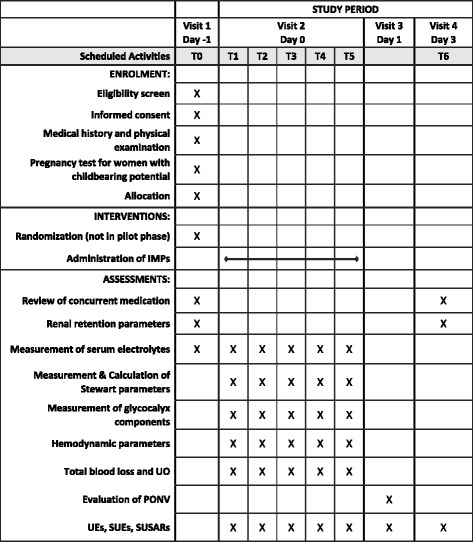
Patient schedule of activities according to the Standard Protocol Items: Recommendations for Interventional Trials (SPIRIT) figure. *SID* Strong Ion Difference, *SUEs* serious unexpected events, *SUSARS* severe unexpected serious adverse event, *UE* unexpected events, *UO* urinary output

This is a single-centre, phase II clinical trial in patients undergoing major abdominal surgery (Fig. 1). The study follows a prospective, controlled and open design. Initially, the study will begin with a pilot phase of six patients followed by a randomization phase. An overview of the patients’ schedule of activities according to the Standard Protocol Items: Recommendations for Interventional Trials (SPIRIT) figure is provided in Fig. 2.

### Pilot phase

The first six patients will receive Jonosteril® together with sodium glycerophosphate (IMP1/IMP2 protocol) in concentrations ranging from 1 to 6 mmol/l (Na^+^: 139–149 mmol/l) to evaluate the most suitable concentration for the following randomization phase. We will test a concentration of 4 mmol/l phosphate (= 2 ml sodium glycerophosphate per 500 ml Jonosteril®) initially. This solution will contain 145 mmol/l sodium. Repeated measurements of serum phosphate levels will shed light on the efficacy of the measure. In the case that serum phosphate levels do not remain stable within the normal range, we will gradually adapt the dose until stable levels are achieved. Patients will not receive a concentration higher than 6 mmol/l since then the sodium levels would surpass the maximum threshold value.

### Randomization phase

After the pilot phase, in which the most suitable phosphate concentration is determined, a total of 42 patients, 21 per group, will be allocated to one of the two study arms by randomization:

- Jonosteril® and sodium glycerophosphate (IMP1/IMP2) (treatment group)
- Jonosteril® (IMP1) (control group)

All patients will receive the study medication according to their allocation over 120 min after the initiation of general anaesthesia in a maximum dose of 30 ml per ideal bodyweight (IBW = height (in cm) − 100) per hour for losses due to insensible perspiration, urinary output and extracellular deficits due to the fasting state as well as five times the estimated blood loss [32, 33]. The rationale for the use of the amount of fluids is based on previous studies and the demand-oriented current clinical practice. This is in concordance with previous studies using similar regimes. The recruited patients have a higher fluid demand [32, 33]. Hofmann-Kiefer et al. showed that a maximum of 30 ml/kg/bodyweight was necessary to keep patients hemodynamically stable and distinct acid-base alterations were described. Over the time course of 120 min, the patients likely suffer from a blood loss of 1000 ml or more. According to current clinical practice, a blood loss should be replaced in a ratio of 4–5:1 with crystalloids [33]. Taken together, our approach using 30 ml/kg/ideal bodyweight seems reasonable for this patient cohort. Measurements will take place every 30 min: baseline (T1), 30 min (T2), 60 min (T3), 90 min (T4) and 120 min (T5) and parameters will be documented by the investigators in the Case Report Forms (CRF) of each participant. An overview of the parameters assessed in this study and their respective time points is provided in Table 1.

**Table 1:** Parameters measured at the distinct time points

Measurement	Time point
Haemodynamic values	
Norepinephrine dose (mg/h)	T1–T5
Heart rate (min^-1^)	T1–T5
Systolic blood pressure (mmHg)	T1–T5
Diastolic blood pressure (mmHg)	T1–T5
Central venous pressure (mmHg)	T1–T5
Stroke volume variation (%)	T1–T5
Cardiac output (ml/min)	T1–T5
Blood loss (ml)	T1–T5
Haemoglobin (g/dl)	T1–T5
Haematocrit (%)	T1–T5
Urine volume (ml)	T1–T5
Crystalloid administered (ml)	T1–T5
Serum electrolytes	
[PO_4_^3-^] ionized (mmol/l)	T0–T5
[PO_4_^3-^] (mg/dl)	T0–T5
[Na^+^] (mmol/l)	T1–T5
[K^+^] (mmol/l)	T1–T5
[Cl^−^] (mmol/l)	T1–T5
[Mg^2+^] (mmol/l)	T1–T5
[Ca^2+^] (mmol/l)	T1–T5
Osmolality (mosmol/l)	T1–T5
Renal function	
Serum creatinine (mg/dl)	T0 and T6
Serum urea nitrogen (mg/dl)	T0 and T6
GFR calculated (ml/min)	T0 and T6
Acid-base values	
Anion gap (mmol/l)	T1–T5
HCO3^-^ (mmol/l)	T1–T5
BE (mmol/l)	T1–T5
pH	T1–T5
PaCO_2_ (mmHg)	T1–T5
Stewart acid-base parameters	
[A^-^] (mmol/l)	T1–T5
Strong Ion Difference (mmol/l)	T1–T5
Effective Strong Ion Difference	T1–T5
Strong Ion Gap (mmol/l)	T1–T5
Albumin (mg/dl)	T1–T5
Lactate (mmol/l)	T1–T5
Urine	
[Urinary Na^+^] (mmol/l)	T1 and T5
[Urinary K^+^] (mmol/l)	T1 and T5
[Urinary Cl^−^] (mmol/l)	T1 and T5
[Urinary Mg^2+^] (mmol/l)	T1 and T5
[Urinary Ca^2+^] (mmol/l)	T1 and T5
[Urine osmolality] (mosmol/l)	T1 and T5
[Urinary phosphate] (mmol/l)	T1 and T5
Glycocalyx components	
Syndecan-1	T1–T5
Hyaluronic acid	T1–T5

*BE* base excess, *GFR* glomerular filtration rate

### Anaesthesiologic management and postoperative care

All patients will receive anaesthesiologic management according to the institution’s standard. This involves a thoracic epidural anaesthesia in combination with general anaesthesia and the placement of a central venous line and an arterial catheter for haemodynamic monitoring. In case of contraindications to neuroaxial procedures patients will receive general anaesthesia and, postoperatively, patient-controlled analgesia with piritramide. Anaesthesia will be induced with propofol (2 mg/kg), sufentanil (0.4 mg/kg) and rocuronium (0.6 mg/kg) and maintained with propofol and remifentanil or sevoflurane in patients with certain conditions (e.g. cardiopulmonary diseases). The pulmonary ventilation will be standardized in all patients using the volume-controlled mode in order to keep the paCO_2_ at 40 ± 3 mmHg as to have as little influence on acid-base balance as possible (controls are performed using blood gas analysis). The epidural anaesthesia will be continued for at least 3 days and will be combined with nonsteroidal anti-inflammatory drugs. Intraoperative hemodynamic monitoring will be performed using a PulsioFlex monitor with ProAQT® sensor (PULSION Medical Systems SE, Germany). The observation period will be 120 min after initiation of anaesthesia although surgery will take longer in most cases. The administration of the study drug, e.g., sodium glycerophosphate, as well as study-specific blood draws, will take place only during the 120 min of the procedure. Afterwards, the anaesthetist responsible for the patient will continue according to clinical standards. All patients will be transferred from the operating room to the ICU for postoperative surveillance and will be visited by in-hospital postoperative pain management service staff daily.

### Trial population and selection criteria

Patients are identified based on their diagnosis and scheduled surgery at the department of gynaecology. We recruit only female patients who are scheduled for major abdominal surgery, i.e. laparotomy. By routine, these patients will undergo placement of a central venous line and an arterial line. Laparoscopic or transvaginal approaches are not included. We screen eligible patients for study participation according to the predefined inclusion and exclusion criteria and provide information regarding the trial during a pre-operatory orientation interview with one of the investigators of the team. We will obtain informed consents from all participants of the study. In addition to informed consent regarding the participation in the study, the patient will be asked to sign a separate informed consent for the frozen storage and possible use of blood samples for scientific purposes to address comments of reviewers during the publication phase. The latter consent is independent of participation in the study. Patients receive complete information about the trial and enough time to consider participation.

#### Inclusion criteria

Subjects must meet the following inclusion criteria to be eligible for enrolment:

- Female patients (ASA Classification I–III) who are scheduled for a major abdominal surgical procedure under general anaesthesia with routinely planned placement of a central venous and an arterial catheter and who have given written informed consent
- Age ≥18 years

#### Exclusion criteria

Subjects showing the following exclusion criteria cannot be included in the trial:

- Participation in another clinical trial
- Patients who are not personally able to give their informed consent
- Patients at a childbearing age without using contraceptives
- Acute or chronic renal failure (glomerular filtration rate <60 ml/min)
- Patients suffering from an acid-base-disturbance (i.e. severe acidosis)
- Patients suffering from acid-base disturbances caused by SIRS or sepsis
- Pregnancy or lactation period (pregnancy testing will be executed at least 1 day prior to the surgical procedure)
- Oedema, hypertonic dehydratation, hyperhidratation
- Hyperphosphata emia, hypernatraemia, hypocalcaemia (according to the standard normal laboratory values of the authors’ institution)
- A known hypersensitivity against the test drugs and/or their components
- A known history or active abuse of alcohol and/or drugs

### Randomization

INPADS GmbH performs the allocation of patients to one of the two study arms using simple, balanced randomization via random numbers. A computer-generated number between 0 and 1 is subsequently analysed as to whether it lies above 0.5 (allocation to IMP1) or below 0.5 (allocation to IMP1 and IMP2 combined). The randomization procedure is executed for all patients after the pilot phase undergo the randomization procedure and a list is generated prior to first patient’s first visit (FPFV).

### Statistical evaluation

#### Power considerations

This study aims to show that [A^−^] can be stabilized by applying IMP1/IMP2 in a combined approach. In a previous investigation of our group, [A^−^] was reduced by 2.67 ± 2.4 mmol/l in patients who had received 4000 ml of Jonosteril® over 120 min [22]. We expect that we can counteract the decrease in [A^−^] up to 60% when using the IMP1/IMP2 protocol. This will presumably result in an average difference in [A^−^] of 1.602 mmol/l in the treatment group. Furthermore, we assume that [A^−^] will be more stable over all time points. As a consequence, the range of [A^−^] will be lower in the treatment compared to the control group. Therefore, we expect that the standard deviation will rather be 1.8 mmol/l than 2.4 mmol/l (estimated reduction of 30%) [22].

#### Sample size calculation

We calculated the sample size using the statistic software SAS (SAS Institute GmbH, Heidelberg, Germany). This study has an explorative character and is supposed to deliver first-ever data. There is currently no comparative data available for the medication group or time intervals considered. For the control group regimen, there is data available from a previous study [22] for two time points (60 min and 120 min after exposure). Therefore, we considered the deviation of [A^−^] at the time point T5 (120 min) compared to baseline T1 (0 min) applying a *t* test. We expect normally distributed data since the control group data derived from the previous study showed the same characteristics [22]. We set the level of significance to 5% and the power to 80% resulting in 21 patients per group. With the addition of swix patients for the pilot phase, the total number of patients is 48.

#### Analysis procedure

The primary objective of the study is to show that during the observation period of 120 min the mean values of [A^−^] display a lower deviation in the treatment group when compared to the control. We will evaluate normal distribution using the Kolmogorov-Smirnov test. To test for differences between two study groups with normally distributed data, a two-sample *t* test will be used. In order to evaluate differences of [A^−^] within one study group over time, we will perform a RM-ANOVA with a *t* test for paired samples as post hoc. We will analyse data that does not follow a normal distribution using the Friedman test. Level of significance for all calculations is defined at 5%. We will analyse secondary parameters descriptively and calculate mean, number, minimum, maximum, 1st quartile, 3rd quartile, mean standard error and standard deviation (SD) (method of aggregation: continuous). Further analyses and testing procedures can be defined at a later stage. The primary statistical analysis is based on the “intention-to-treat” principle. Safety data (UE, SUE, SUSARs) will be analysed descriptively in all groups at least every 12 months.

#### Data handling and dropouts

INPADS GmbH will perform data management according to the DEGL and DMP. All patients who drop out will be listed, followed-up and monitored. We will document the reason for dropping out and all data registered until then will be included in the analysis. After inclusion of six patients, we will perform an interim analysis. Although dropouts will be very unlikely, due to the design of the study, we will replace the dropout in the case of such an event. Strategies to improve adherence to protocols involve the participation of an investigator (anaesthesiologist) who is responsible for the trial during the initiation of anaesthesia and in the observation period as well as for the completion of checklists in similar format of the CRF. Since patients are under anaesthesia during the intervention, other strategies, such as patient focused techniques, are not intended.

### Ethics and Good Clinical Practice

The trial will be conducted in accordance with the Clinical Trials Directive 2001/20/EC of the European Parliament and of the Council, the International Conference on Harmonization guidance regarding Good Clinical Practice (ICH-GCP E6 R1), the relevant national regulations and the Declaration of Helsinki. Monitoring is independent from the sponsor and competing interests. If external auditing is demanded by an authority, it will be independent from investigators and sponsor. Any modifications to the protocol will be, and have been, immediately communicated to all responsible authorities.

## Discussion

Although there is currently a broad variety of so-called balanced crystalloid preparations available, none of them is ideal and capable of stabilizing the patient’s acid-base balance. This is due to the nature of their composition. Stewart’s concept of acid-base homeostasis may provide a new approach to improve the daily clinical routine of fluid administration. By administering sodium glycerophosphate pre-emptively, it is hypothesized that stable values of [A^−^] can be maintained in patients in the need for larger amounts of fluids due to illness or type of surgery. Furthermore, by supplementing phosphate perioperatively, a frequently occurring hypophosphatemia can be avoided. This trial will increase our knowledge in the applicability of Stewart’s concept of acid-base as well as in the field of perioperative fluid substitution, an area that has a broad applicability in medicine and is directly linked to the patients’ outcome.

### Trial status

Recruiting: participants are currently being recruited and enrolled.

### SPIRIT guidelines

The PALANCE study protocol was written in accordance with the Standard Protocol Items: Recommendations for Interventional Trials (SPIRIT). A completed SPIRIT Checklist (Additional file 1) and a SPIRIT figure (Fig. 2) has been included in this manuscript.

## Additional file

Additional file 1: SPIRIT Checklist. (PDF 175 kb)

## Abbreviations

AE: Adverse event
ASA: American Society of Anaesthesiologists’ performance status classification system
CRF: Case Report Form
CRO: Contract Research organizations
DEGL: Data entry guideline
DMP: Data management plan
ECG: Electrocardiography
GFR: Glomerular filtration rate
Hb: Haemoglobin
ICU: Intensive care unit
IMP: Investigational medicinal product
ITT: Intention-to-treat
IV: Intravenous
SAE: Serious adverse event
SDP: Sponsor-delegated person
SD: Standard deviation
SUE: Severe unexpected event
SUSAR: Severe unexpected serious adverse event
SVV: Stroke volume variation
UE: Unexpected event

## References

[1] Holte K, Kehlet H (2006). Fluid therapy and surgical outcomes in elective surgery: a need for reassessment in fast-track surgery. J Am Coll Surg.

[2] Jacob M, Chappell D, Rehm M (2007). Clinical update: perioperative fluid management. Lancet.

[3] O’Malley CM, Frumento RJ, Hardy MA, Benvenisty AI, Brentjens TE, Mercer JS (2005). A randomized, double-blind comparison of lactated Ringer’s solution and 0.9% NaCl during renal transplantation. Anesth Analg.

[4] Guidet B, Soni N, Della Rocca G, Kozek S, Vallet B, Annane D (2010). A balanced view of balanced solutions. Crit Care.

[5] McFarlane C, Lee A (1994). A comparison of Plasmalyte 148 and 0.9% saline for intra-operative fluid replacement. Anaesthesia.

[6] Brill SA, Stewart TR, Brundage SI, Schreiber MA (2002). Base deficit does not predict mortality when secondary to hyperchloremic acidosis. Shock.

[7] Chowdhury AH, Cox EF, Francis ST, Lobo DN (2012). A randomized, controlled, double-blind crossover study on the effects of 2-L infusions of 0.9% saline and plasma-lyte (R) 148 on renal blood flow velocity and renal cortical tissue perfusion in healthy volunteers. Ann Surg.

[8] Wilkes NJ, Woolf R, Mutch M, Mallett SV, Peachey T, Stephens R (2001). The effects of balanced versus saline-based hetastarch and crystalloid solutions on acid-base and electrolyte status and gastric mucosal perfusion in elderly surgical patients. Anesth Analg.

[9] Shaw AD, Schermer CR, Lobo DN, Munson SH, Khangulov V, Hayashida DK (2015). Impact of intravenous fluid composition on outcomes in patients with systemic inflammatory response syndrome. Crit Care.

[10] Barmparas G, Liou D, Lee D, Fierro N, Bloom M, Ley E (2014). Impact of positive fluid balance on critically ill surgical patients: a prospective observational study. J Crit Care.

[11] de Oliveira FS, Freitas FG, Ferreira EM, de Castro I, Bafi AT, de Azevedo LC (2015). Positive fluid balance as a prognostic factor for mortality and acute kidney injury in severe sepsis and septic shock. J Crit Care.

[12] Hasselbalch KA (1917). Die Berechnung der Wasserstoffzahl des Blutes aus der freien und gebundenen Kohlensäure desselben, und die Sauerstoffbindung des Blutes als Funktion der Wasserstoffzahl. Biochem Z.

[13] Astrup P, Joergensen K, Andersen OS, Engl K (1960). The acid-base metabolism, a new approach. Lancet.

[14] Emmett M, Narins RG (1977). Clinical use of the anion gap. Medicine.

[15] Stewart PA (1981). How to understand acid-base. A quantitative acid-base primer for biology and medicine.

[16] Stewart PA (1983). Modern quantitative acid-base chemistry. Can J Physiol Pharmacol.

[17] Seifter JL (2015). Integration of acid-base and electrolyte disorders. N Engl J Med.

[18] Rehm M, Conzen PF, Peter K, Finsterer U (2004). The Stewart model. “Modern” approach to the interpretation of the acid-base metabolism. Anaesthesist.

[19] Figge J, Rossing TH, Fencl V (1991). The role of serum proteins in acid-base equilibria. J Lab Clin Med.

[20] Morgan TJ, Venkatesh B (2003). Designing “balanced” crystalloids. Crit Care Resusc.

[21] Morgan TJ, Venkatesh B, Hall J (2002). Crystalloid strong ion difference determines metabolic acid-base change during in vitro hemodilution. Crit Care Med.

[22] Hofmann-Kiefer KF, Chappell D, Kammerer T, Jacob M, Paptistella M, Conzen P (2012). Influence of an acetate- and a lactate-based balanced infusion solution on acid base physiology and hemodynamics: an observational pilot study. Eur J Med Res.

[23] Zazzo JF, Troche G, Ruel P, Maintenant J (1995). High incidence of hypophosphatemia in surgical intensive care patients: efficacy of phosphorus therapy on myocardial function. Intensive Care Med.

[24] Buell JF, Berger AC, Plotkin JS, Kuo PC, Johnson LB (1998). The clinical implications of hypophosphatemia following major hepatic resection or cryosurgery. Arch Surg.

[25] Cohen J, Kogan A, Sahar G, Lev S, Vidne B, Singer P (2004). Hypophosphatemia following open heart surgery: incidence and consequences. Eur J Cardiothorac Surg.

[26] Salem RR, Tray K (2005). Hepatic resection-related hypophosphatemia is of renal origin as manifested by isolated hyperphosphaturia. Ann Surg.

[27] George R, Shiu MH (1992). Hypophosphatemia after major hepatic resection. Surgery.

[28] Giovannini I, Chiarla C, Nuzzo G (2002). Pathophysiologic and clinical correlates of hypophosphatemia and the relationship with sepsis and outcome in postoperative patients after hepatectomy. Shock.

[29] Geerse DA, Bindels AJ, Kuiper MA, Roos AN, Spronk PE, Schultz MJ (2010). Treatment of hypophosphatemia in the intensive care unit: a review. Crit Care.

[30] Hsu HJ, Wu MS (2008). Extreme hyperphosphatemia and hypocalcemic coma associated with phosphate enema. Intern Med.

[31] Cockcroft DW, Gault MH (1976). Prediction of creatinine clearance from serum creatinine. Nephron.

[32] Jacob M, Chappell D, Hofmann-Kiefer K, Helfen T, Schuelke A, Jacob B (2012). The intravascular volume effect of Ringer’s lactate is below 20%: a prospective study in humans. Crit Care.

[33] Rehm M, Hulde N, Kammerer T, Meidert AS, Hofmann-Kiefer K (2017). State of the art in fluid and volume therapy: a user-friendly staged concept. English version. Anaesthesist.

